# Feasibility of home-based HIV counselling and testing and linking to HIV services among women delivering at home in Geita, Tanzania: a household longitudinal survey

**DOI:** 10.1186/s12889-019-8111-4

**Published:** 2019-12-30

**Authors:** Juma Adinan, Bridgit Adamou, Caroline Amour, Aisa Shayo, Paulo Lino Kidayi, Levina Msuya

**Affiliations:** 1 AMO School KCMC, P.O.Box 2316, Moshi, Tanzania; 2 Kilimanjaro Christian Medical Centre, Community Health department, Moshi, Tanzania; 3 Kilimanjaro Christian Medical University College, Institute of Public Health, Moshi, Tanzania; 4 University of North Carolina at Chapel Hill, Chapel Hill, USA; 5 Kilimanjaro Christian Medical Centre, Paediatric and Child Health department, Moshi, Tanzania; 6 Kilimanjaro Christian Medical University College, Faculty of Nursing, Moshi, Tanzania

**Keywords:** PMTCT, Home-based HIV Counselling and testing, Linkage to care, Women delivering at home, Paediatric HIV, Home deliveries

## Abstract

**Background:**

Substantial number of women who deliver at home (WDH) are not captured in prevention of mother-to-child transmission (PMTCT) services. This delays HIV infection detection that negatively impacts endeavours to fight the HIV pandemic and the health of mothers and children. The study objective was to determine the feasibility of home-based HIV testing and linking to care for HIV services among WDH in Geita District Council, Tanzania.

**Methods:**

A longitudinal household survey was conducted. The study involved all mentally-able women who delivered within 2 years (WDTY) preceding the survey and their children under the age of two. The study was conducted in Geita District Council in Geita Region, Tanzania from June to July 2017. Geita is among the region with high HIV prevalence and proportion of women delivering at home.

**Results:**

Of the 993 women who participated in the study, 981 (98.8%) accepted household-based HIV counselling and testing (HBHCT) from the research team. HIV prevalence was 5.3% (52 women). HBHCT identified 26 (2.7%) new HIV infections; 23 (23.4%) were those tested negative at ANC and the remaining three (0.3%) were those who had no HIV test during the ANC visit. Among the 51 HIV+ women, 21 (40.4%) were enrolled in PMTCT services. Of the 32 HIV+ participants who delivered at home, eight (25.8%) were enrolled in the PMTCT compared to 100% (13/13) of the women who delivered at a health facility.

**Conclusion:**

HBHCT uptake was high. HBHCT detected new HIV infection among WDH as well as seroconversion among women with previously negative HIV tests. The study findings emphasize the importance of extending re-testing to women who breastfeed. HBHCT is feasible and can be used to improve PMTCT services among WDH.

## Background

Despite major progress in implementing interventions for PMTCT in Sub-Saharan Africa, rates of new paediatric HIV infections remain unacceptably high, contributing to over 10% of new HIV infections globally and 15% of all HIV related mortality [1] .

The PMTCT program effectively reduces mother-to-child transmission of HIV. Women are enrolled in the program at the health facility, either at an antenatal care (ANC) visit, or when they come for delivery. In Tanzania, only around 60–70% of pregnant women receive HIV counselling and testing during ANC [2–5] and 49% of women do not deliver at a health facility [6]. Low testing uptake and home deliveries affects PMTCT enrolment and undermines the achievement of global HIV targets [7].

Household-based HIV counselling and testing has been shown in different countries to be effective. HBHCT is an in-home HIV testing service where a person is referred to a health facility for subsequent care if s/he tests positive. Though there are documented drawbacks and conflicting results, HBHCT’s effectiveness is widely accepted [8–14]. HBHCT has shown positive impact not only in detecting HIV new infections among pregnant and postpartum women [15] but also in influencing men to test [16–19] and participate in PMTCT services in Africa. Despite the introduction of home-based testing in Tanzania in 2013, HBHCT has never been evaluated in Tanzania for its effectiveness.

To achieve global HIV 90–90-90 [20] targets requires well-thought out and piloted strategies to detect HIV infection early and link HIV-positive individuals to care. Under the expanding PMTCT outcomes (EPO) project, we sought to determine the feasibility of home-based HIV testing and linking to HIV services among WDH in Geita District Council, Tanzania.

## Methods

### Study design and setting

**Fig. 1:**
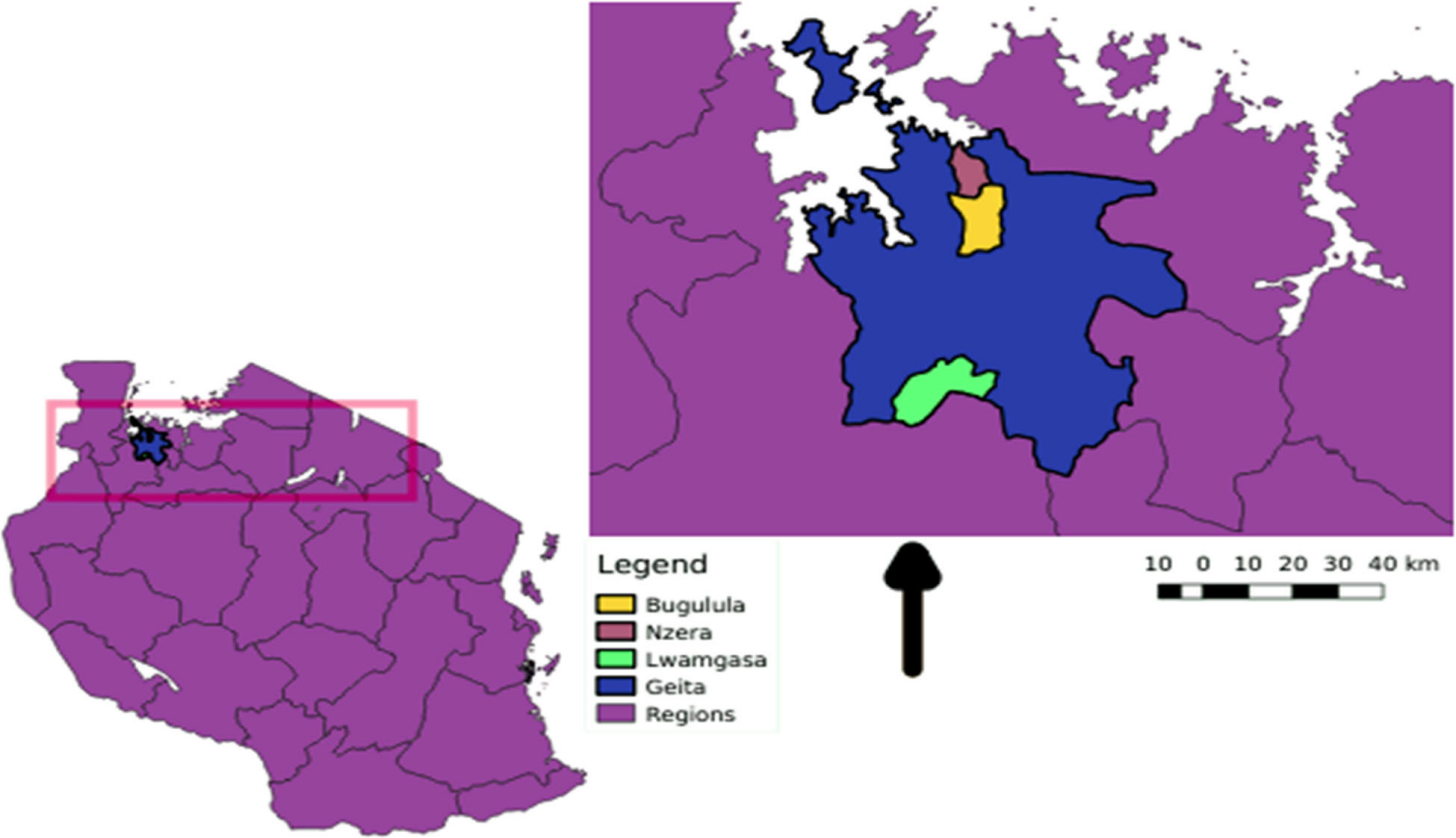
Map of Geita Region showing the wards participated in the study: Bugulula, Yellow; Nzera, Red and Rwamgasa, Green. The map was generated by using QGIS 3.8

The aim of the study was to determine the feasibility (i.e., perception plus uptake of HBHCT) of home-based HIV testing and linking to HIV services among WDH in Geita District Council, Tanzania. We employed longitudinal household survey conducted in Geita District Council, a rural area in Geita Region, Tanzania (Fig. 1). Three representative wards were selected: Nzera, Bugulula, and Rwamgasa. Socio-economic activities in Geita are mainly small-scale farming, business and mining. The study was conducted from June to July 2017.

In Geita Region, more women deliver at home (52%) than the national average [21], but the adult HIV prevalence (3–6%) is on par with the national average (4.7%) [6].

### Sample size and sampling technique

The study involved both WDH and women who delivered at a health facility within 2 years preceding the survey along with their children under the age of two. These two groups of women were compared in terms of acceptability and perception of household-based HIV counselling and testing (HBHCT). With no HIV prevalence data among WDTY in Geita District Council, the HIV prevalence of Geita Region was taken as a proxy estimate. We calculated a 5% margin of error and 95% confidence interval, giving an initial sample size estimation of 844. A standard 10% non-response rate was added to the sample to increase the minimum sample size to 928 WDTY. In total, 993 women participated in the study, along with 52 children whose mothers tested HIV-positive or refused an HIV test.

### Selection of households

The unit of analysis was WDTY. A multistage cluster sampling technique was used to select households to be involved in the study. Three representative wards were selected: Nzera, Bugulula, and Rwamgasa. Within each ward, three villages were randomly selected and surveyed (except for Rwamgasa, where two villages were sampled). Two hamlets were selected from each village using simple random selection. The final stage was the systematic random selection of households. To identify households with WDTY, we asked household occupants. Once the household with WDTY was identified, we enrolled only one WDTY per household. If the household had more than one qualifying participant, we used random selection. A piece of paper was prepared based on the number of qualifying participants in the household. Each eligible individual randomly picked a piece of paper. Whoever picked the piece of paper that said “yes” was recruited into the study. Data collectors bypassed household without WDTY, they approached the subsequent house in the determined direction.

### Community sensitization

To sensitize the community, we engaged local leaders in the process of implementing home-based HIV testing. The role of the local leaders were to inform the community of the project and to clear myths the community might have had about the goal of HIV testing.

Local leaders involved were ward executive officers from all participating wards, village chairmen and village secretaries from all participating villages.

We conducted a one-day workshop with the following objectives: 1) introduce the project and project team; 2) explain the importance of the project and findings; 3) describe how the project will be conducted; 4) explain the mobilization roles the leaders were going to play; and 4) share strategies of how to make the project successful based on people’s experiences, for example, when to visit a house and other cultural matters.

Local leaders passed information to all households alerting the study population a day or two before we visited the household.

### Data sources, collection and referral

#### Interview

We used pretested questionnaire by Adeleke et al. [22]. The questionnaire contained structured and open-ended questions. Questions covered a range of topics including socio-demographic characteristics of the mothers and their children, mother’s birth history which included ANC visits, PMTCT information (knowledge of PMTCT, history of HIV, number and interval of HIV tests done at ANC, and whether PMTCT services were provided), risk factors for acquiring HIV infection, household possessions for assessing wealth index, contextual factors that would influence healthcare-seeking behaviour, and questions related to HIV/ PMTCT program interventions. We collected qualitative data to gain an in-depth understanding of the reasons for not having an HIV test during ANC and reasons for not enrolling in PMTCT for the mothers who were aware of their HIV-positive status. Along with participant’s contacts, we collected three contacts of participants’ close people (relatives, friends or colleagues). These contacts were used to trace participants in case a break of communication between investigators and participant occurred.

### Home-based HIV counselling and testing

Antibody-based HIV (HIV rapid) tests were used to determine the HIV-serostatus of study participants [23, 24]. HBHCT was offered to WDTY regardless of the time lapse since their last test. HIV tests were offered to those who consented to testing and to the children ages 9–23 months whose mothers tested HIV-positive as part of the study-administered HBHCT. We followed both the national HIV algorithm [25] and manufacturer directives for testing and results interpretation.

### Referral and follow-up phone interview

**Fig. 2:**
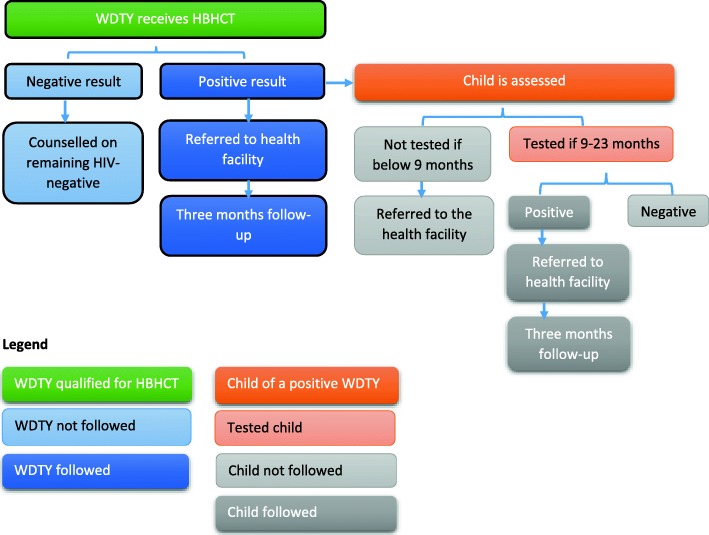
Study Algorithm showing eligible participants and their level of participation into the study

We referred to a health facility for testing, care, and treatment, as necessary, mothers who had never tested for HIV previously and refused a study-administered HIV test; mothers who knew their HIV-positive status but were not enrolled in PMTCT; and mothers who were identified as HIV-positive by the study. Participants who were referred to a health facility for subsequent care were followed-up (Fig. 2) by phone three times (at 1 month, 2 months, and 3 months) to assess if they followed through on their referral, their HIV serostatus, and, if appropriate, uptake of PMTCT services. A successful referral was assessed at the last follow-up.

### Definition of variables

Feasibility of HBHCT in detecting HIV was defined as an acceptance rate of ≥90% of home-based HIV testing and an acceptance rate of ≥90% of receiving results by women delivering at home/ within 2 years.

Feasibility in linking to care was defined as ≥50% of the referred individuals reporting a completed referral.

### Data quality assurance and analysis

We used Kobotoolbox® for data collection. The study’s primary outcome was the feasibility of HBHCT. The secondary outcome was the successful link to care of HIV positive WDH.

We transferred data from Kobotoolbox® to Stata Version 15 for cleaning, processing, and analysis. The analysis took into consideration the clustering effect. Hamlets were clusters in this study. We used population statistics to generate post-sampling weight to account for the ward-level variance in sample size.

A *p*-value of less than 0.05 was considered for statistical significance independence during comparison between WDH and those delivered at the health facilities.

## Results

### Background characteristics of participants

In total, 993 WDTY in Geita District Council participated in the study. The median age was 25 years, with the largest proportion in the 16- to 24-year age band. Most resided in Rwamgasa (47.7%), had a primary education (39.6%), were married or in-union (89.1%), and had one or two children (43.6%). Fewer than 10% had health insurance coverage, and most (92.5%) did not consider the health facility they usually access for health services as being far from their home. Nearly all the participants (946; 95.2%) attended at least one ANC visit during their last pregnancy. Among these women, 81.7% (773) were tested for HIV during ANC attendance, with 26 (3.3%) testing positive. More than half (56.9%) of the participants had a home birth for their last delivery (Table 1).

**Table 1:** Participants’ background characteristics (*n* = 993). Un weighted and weighted Per cent distribution of participant’s background characteristics, EPO project 2017

	Not weighted		Weighted	
	No.	%	No.	%
Age				
16–24	435	43.80	478/479	48.20
25–35	413	41.60	374	37.70
36–51	145	14.60	140	14.10
Education ^a^				
No formal education	342	34.50	324	32.60
Did not complete primary school	206	20.80	217	21.80
Primary education	402	40.50	393	39.60
Secondary education	41	4.10	54	5.40
Higher education	1	0.10	5	0.50
Marital status^a^				
Single	49	5.00	46	4.70
Married/In union	851	87.10	870	89.10
Divorced	77	7.90	61	6.20
Ward of residence				
Nzera	439	44.20	223	22.50
Bugulula	397	40.00	296	29.80
Rwamgasa	157	15.80	473/474	47.70
Occupation				
Small-scale farmers	959	96.58	954	96.06
Self-employed business	29	2.92	36	3.60
Salaried employee	5	0.50	3	0.34
Number of children				
1–2	397	40.00	433	43.60
3–4	264	26.60	262	26.40
5–14	332	33.40	298	30.00
Covered by health insurance^a^				
No	895	90.20	897	90.40
Yes	97	9.80	95	9.60
Is the usual health facility you access far?				
No	858	86.40	918	92.50
Yes	135	13.60	75	7.50
Place of last delivery				
Home	554	55.80	565	56.90
Health facility	439	44.20	428	43.10
Attended ANC in the last pregnancy				
No	36	3.60	47	4.80
Yes	957	96.40	946	95.20
HIV test at ANC visit (*n* = 957/946)				
No	228	23.80	173	18.30
Yes	729	76.20	773	81.70
HIV results at ANC visit (*n* = 729/773)^a^				
Positive	12	1.65	26	3.34
Negative	708	97.12	741	95.87

^a^Missing values

### Uptake of HIV testing services at ANC and HBHCT

Of the 946 (95.2%) participants who visited ANC when pregnant, 767 (81.1%) were tested for HIV. Of those tested, 26 (3.4%) tested positive for HIV.

Nearly all the study participants (981; 99.8%) accepted HBHCT. Specifically, 562 (99.4%) of WDH and 419 (97.9%) of women who delivered at a hospital accepted HBHCT. HBHCT identified 26 (2.7%) new HIV infections among the women; 23 were those who had tested negative at ANC and the remaining three were those who had no HIV test during an ANC visit.

Reasons mentioned for not testing for HIV at an ANC visit included lack of testing kits at the ANC, not being given test results on time, and financial barriers. Reasons for refusing HBHCT included being recently tested for HIV, needing permission from husband, and afraid of receiving a positive test result.

### Prevalence of HIV among participants

**Fig. 3:**
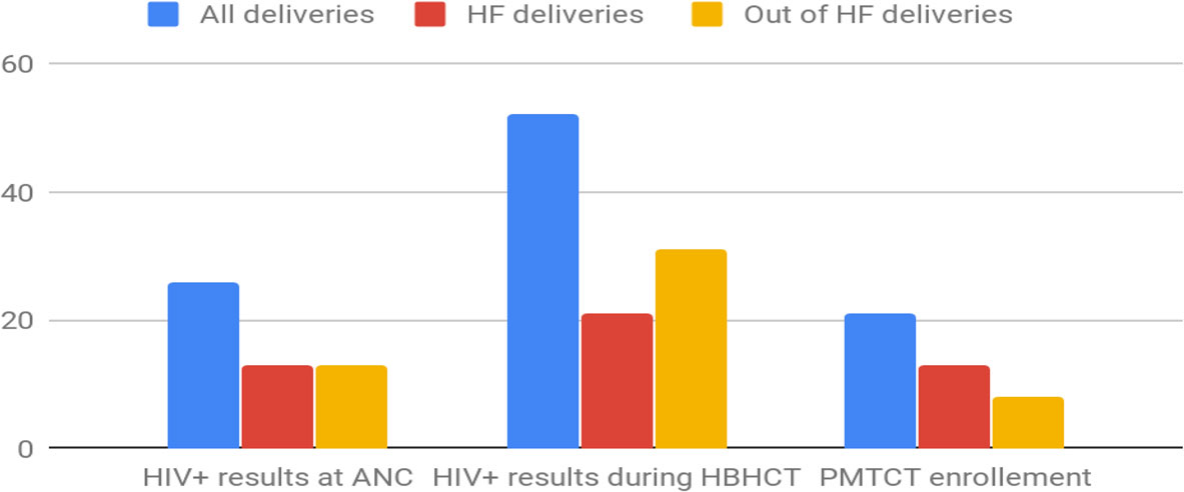
Per cent distribution of HIV prevalence and uptake of PMTCT by places of delivery

The prevalence of HIV among WDTY was 52 (5.3%) [95% CI: 2.1–12.8%]. Thirty one (59.6%) of the HIV-positive participants had delivered at home; among the HIV-positive WDH, 18 (58.1%) were identified during HBHCT. Most of these women were in the 25- to 35-years age category (Fig. 3.).

Twenty-seven of the children delivered by HIV-positive women were tested for HIV. Seven children were HIV-positive. All were children of mothers who were newly detected by HBHCT; six were children of six mothers who had tested negative at ANC, and the one remaining was the child from the mother who had not had HIV test during ANC visit.

Twenty-five children of HIV-positive mothers could not be tested as 24 were below 9 months of age and one was because of mother’s refusal.

### PMTCT uptake among HIV-positive participants

Among the 52 HIV-positive women, 21 were enrolled in PMTCT services. All 13 HIV-positive women who delivered at a health facility and knew their HIV-positive status before the HBHCT were enrolled in PMTCT services, contrary to their 13 WDH counterparts, where only eight were enrolled in PMTCT services (Fig. 3.)

The five WDH who knew their HIV-positive status before the study mentioned fear of family conflicts as the main reason not to enrol in PMTCT services.

### Perception of effectiveness of HBHCT among participants

When participants were asked about their perception of HBHTC services compared to facility-based HIV testing and counselling, most agreed that HBHCT saves time (77.2%), reaches more people (66.7%), and links to PMTCT earlier (59.7%). The differences in perception between Women delivering at facilities and women delivered at home that HBHCT saves time (81.3% Vs 77.2%) and links to PMTCT earlier (64.5% Vs 69.7%) is statistically significant. (Table 2).

**Table 2:** Perception of participants on HBHTC effectiveness, efficiency, and early care for HIV (*n* = 933)

Variables	Facility-based delivery	Home-based delivery	Total	X^2^ (*P*-Values)
	No. (%)	No. (%)	No. (%)	
HBHCT saves time				
Agree	357 (81.3)	410 (74)	767 (77.2)	7.80 (0.020)
Indifferent	52 (11.8)	97 (17.5)	149 (15)	
Disagree	30 (6.8)	47 (8.5)	77 (7.8)	
HBHCT reaches more people				
Agree	305 (69.5)	357 (64.4)	662 (66.7)	
Indifferent	48 (10.9)	78 (14.1)	126 (12.7)	3.26 (0.195)
Disagree	86 (19.6)	119 (21.5)	205 (20.6)	
HBHCT links to PMTCT earlier				
Agree	283 (64.5)	310 (56)	593 (59.7)	
Indifferent	138 (31.4)	220 (39.7)	358 (36.1)	7.65 (0.022)
Disagree	18 (4.1)	24 (4.3)	42 (4.2)	

Per cent distribution and comparison of perception of women who delivered at home versus those delivered at the health facilities on the importance of HBHCT in terms of saving time, coverage and link to PMTCT services earlier, EPO project 2017

### Referrals to health facilities for HIV testing and PMTCT services

The study team referred 43 mothers to health facilities; 12 for testing after denying HBHCT and 31 for subsequent PMTCT services following positive HIV results. Researchers managed follow-up communication with 16 participants and of these, 12 completed the referral. Almost two-thirds (27; 62.8%) of the referred participants were not reachable for different reasons. Of the 12 participants who attended referral, seven were among those referred for testing.

Among the 31 participants who were HIV-positive and referred for subsequent PMTCT care, only five successfully attended referral; of these, four were newly-identified HIV-positive. Two participants gave the excuse that they were travelling and one reported lack of time.

## Discussion

This study was conducted to determine the feasibility of HBHCT for early detection of HIV infection among women up to 2 years postpartum and linking HIV-positive women to subsequent HIV care in Geita District Council, Geita, Tanzania.

The HIV prevalence among the adult study participants was 5%. This prevalence is similar to the HIV prevalence of 3–6% reported by the National Bureau of Statistics (2016) [21]. The most affected were in the 25- to 35-years age category and were residents of Rwamgasa—the ward where mining is the main economic activity. These support findings already reported on risky population groups based on the 25- to 35-years age [21] category living in areas where the main economic activity is mining [26].

The study showed that HBHCT is highly acceptable, with 99% of participants agreeing to be tested. The high acceptability signifies the suitability of HBHCT as a method of early HIV detection among the study population. This finding is similar to those reported in different parts of Africa [11, 27], including Tanzania [12, 28]. However, this study reported very high acceptability of HBHCT compared to other studies, which reported 60–70% acceptance.

High acceptability can be explained by the fact that participants were reached and tested at their homes. The reason we had an increased uptake in the number of women to be tested was due to the fact that the women in this community are predominantly farmers. Hence most of their activities are being done near their homes, hence bringing the service close to home was readily accepted as it does not take them away from their activities. Also the men had a buyin in acceptance of the testing and since it is a predominantly male dominated community, we had a clear passage and an increased uptake in testing.

High acceptability can also be explained by the role played by local leaders in informing the community of the study and the trust that was built with the researchers. Local leaders did community mobilization on the screening services as well as escorting data collectors to households. Research assistants reported community members’ readiness and enthusiasm towards HIV testing regardless of their eligibility criteria. This was evident by the 393 men and other women who did not meet the inclusion criteria who asked for the HIV test.

HBHCT identified half (50%) of the HIV cases in our study population. More so, the study revealed seroconversion among women who tested negative during their last HIV test at ANC. These findings not only emphasize the importance of HBHCT in improving access to HIV services [8] but also the importance of extending re-testing [29, 30] to women who breastfeed to effectively implement PMTCT programs. Seroconversion among pregnant women have previously been reported [31, 32]. These findings are similar to those reported in Malawi [12] and Tanzania [30].

Only about two-thirds (8/13) of the WDH who knew their positive HIV status before HBHCT were enrolled in PMTCT services compared to 100% (13/13) of the women who delivered at a health facility. These findings emphasize the importance of health facility deliveries over home deliveries and the importance of scaling up PMTCT services to women who deliver at home.

The strength of the study lies in the rigorous planning, data collection, and analysis. The study was able to show the feasibility of HBHCT in terms of acceptability and uptake among women who are within 2 years postpartum. The study managed to detect the majority of HIV-positive WDH who were unaware of their serostatus and linked them to PMTCT services.

However, the study’s limitations are important to be noted. The study could not detect the effectiveness of HBHCT in linking HIV-positive women to PMTCT and subsequent HIV care. Also, the study suffered from communication barrier challenges that caused loss to follow-up. We used mobile phones to track participants and could only contact less than half of the referred participants. We could not test children younger than 9 months as we used antibody-based HIV rapid test. In addition to the limitation in the designing, HBHCT was offered to any women delivering at home within 2 years. So, some children might not have benefited from the intervention as the time for the PMTCT initiation might have had passed. Finally, we studied a rural population, so the study findings cannot be representative of an urban population.

These findings are important to policy makers and other stakeholders working to address HIV burden. With the increased evidence of higher risk of infection among pregnant and postpartum women [33] and poor implementation of repeat HIV testing during pregnancy [34, 35], the findings show the feasibility [36] and cost-effectiveness [18] of home-based HIV counselling and testing. Beneficiaries of this study are national and sub-national stakeholders working to control and eradicate HIV, such as Ministries responsible for health, district-level health managers, and implementing partners; researchers in the field of community-based services, HIV, and health systems; and donors of HIV interventions.

## Conclusion

HBHCT uptake was high. HBHCT detected new HIV infection among WDH as well as seroconversion among women with previously negative HIV tests. The study findings emphasize the importance of extending re-testing to women who breastfeed. HBHCT can be used to improve PMTCT services among WDH.

## Data Availability

The datasets analysed during the current study are available from the corresponding author on reasonable request.

## Abbreviations

ANC: Antenatal care
HBHCT: Home-based HIV counselling and testing
HIV: Human immune deficiency virus
PMTCT: Prevention of mother to child transmission
WDH: Women who delivered at home
WDTY: Women who delivered within 2 years
